# Knowledge and practices of dog and cat owners in Mainland Portugal regarding fleas, flea-borne pathogens, and their management

**DOI:** 10.1186/s13071-025-06876-y

**Published:** 2025-07-04

**Authors:** Rafael Rocha, Teresa Novo, Carla Maia

**Affiliations:** Global Health and Tropical Medicine, GHTM, LA-REAL, Instituto de Higiene e Medicina Tropical, IHMT, Universidade NOVA de Lisboa, Lisbon, Portugal

**Keywords:** Flea, Dog, Cat, Knowledge, Practices, Flea-borne pathogens, Zoonosis, Portugal

## Abstract

**Background:**

Fleas are the most common ectoparasites of dogs and cats worldwide, causing dermatological problems and transmitting pathogens, some of zoonotic concern.

**Objective:**

To assess the knowledge and practices (KP) of companion animal owners in Mainland Portugal regarding fleas, flea-borne pathogens, and measures for their treatment and prevention.

**Methods:**

A cross-sectional study conducted between March 2022 and March 2023 targeted dog and/or cat owners from the five Mainland Portuguese NUTS2 (Nomenclature of Territorial Units for Statistics) regions. Participants answered a self-administered sociodemographic and KP questionnaire. Individual KP scores were calculated based on predefined grades.

**Results:**

The study included 550 participants: 212 exclusively dog owners, 158 exclusively cat owners, and 180 owners of both species. The median age was 40 years, and 69.9% were female. Veterinarians were the primary source of information about fleas and flea-borne pathogens. Over 10% of participants were unable to identify adult fleas’ characteristics. Most participants (90.3%) identified the environment outdoors as the most common source of infestation, while 54.4% mentioned contact with other animals. While 81.9% recognised that fleas transmit pathogens, only 12.7% could name specific pathogens; the flea bite was the most identified route of transmission. Most participants (87.6%) were aware that fleas parasitising pets could also parasitise humans. The most common flea treatment schedule was every 3–4 months, primarily to prevent infestation, while cleaning and vacuuming house/animal resting places were the preferred control measures. Multivariate analysis revealed that younger participants (≤ 50 years, for fleas), females (for flea-borne pathogens), those with higher education, and residents in the NUTS2 Centro, Área Metropolitana de Lisboa (AML), or Algarve regions had above-median knowledge scores. Higher education, residence in NUTS2 Centro or AML, pet ownership of only cats or dogs, and higher knowledge scores were associated with improved practices.

**Conclusions:**

Most pet owners were aware that fleas transmit pathogens and knew ways to prevent infestations. However, knowledge gaps remain in understanding flea biology and the specific routes of transmission of flea-borne pathogens, which may hinder effective prevention efforts. Veterinarians play a crucial role in educating owners about flea management and prevention of flea-borne infections, aiming at reducing transmission risks to both animals and humans.

**Graphical abstract:**

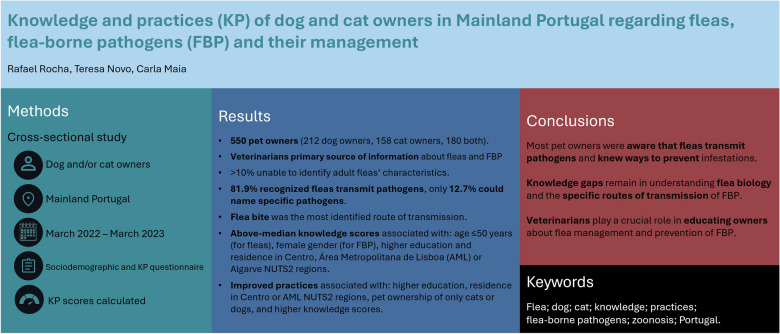

**Supplementary Information:**

The online version contains supplementary material available at 10.1186/s13071-025-06876-y.

## Background

Fleas are among the most relevant parasitic arthropods of veterinary and medical importance worldwide, causing discomfort, anaemia, and allergic reactions known as flea allergic dermatitis. In addition to these direct effects, these blood-feeding insects serve as vectors for multiple pathogens, many of which are zoonotic [1]. The cat flea *Ctenocephalides felis* is considered the most clinically relevant ectoparasite in dogs and cats. This flea species is a competent vector and intermediate host for several zoonotic pathogens, including the bacteria *Rickettsia felis* (the causative agent of flea-borne spotted fever), *Bartonella henselae*, *Bartonella clarridgeiae*, and *Bartonella koehlerae* (the causative agents of cat-scratch disease and endocarditis), as well as the tapeworm *Dipylidium caninum* and the filarial nematode *Acanthocheilonema reconditum*, which cause dipylidiosis and acanthocheilonemiosis, respectively [2–6].

Dogs and cats, the most common pets worldwide [7], provide numerous physiological and mental health benefits to their owners [8]. However, their close interaction with humans and shared environments can potentially increase the opportunities for flea infestation and transmission of flea-borne zoonotic pathogens [1, 6]. In Portugal, 39% of households owned at least one dog and 32% owned at least one cat in 2022, amounting to approximately 2,840,000 dogs and 1,957,000 cats [9]. *Ctenocephalides felis* is the most prevalent flea species infesting both stray and domestic dogs and cats in the country (Pereira et al., personal communication). Other flea species, such as *Ctenocephalides canis*, were found to infest cats and dogs to a much lesser extent, while *Pulex irritans* and *Archaeopsylla erinacei maura* were detected exclusively in dogs (Pereira et al., personal communication). Cases of dipylidiosis, acanthocheilonemiosis, and infections with flea-borne *Bartonella* species have been documented in domestic and stray dogs and/or cats [10–20]. Additionally, human cases of cat-scratch disease have been reported in the country [21–28], and *B. clarridgeiae* and *R. felis* have been detected in fleas [10, 29].

Considering that dogs and cats in Europe are commonly infested with fleas [30], comprehensive guidelines for the effective treatment and control of fleas were elaborated by the European Scientific Counsel for Companion Animal Parasites (ESCCAP; http://www.esccap.org/), aiming not only to safeguard the health of pets but also to protect human health by reducing the risk of zoonotic parasite transmission [31].

Despite the widespread distribution of fleas and the significant burden of flea-borne infections, studies on pet owners’ awareness of these ectoparasites and the zoonotic potential of flea-transmitted pathogens are scarce in Portugal. Existing studies are either focused on parasitic zoonoses or were region-specific or restricted to either dog or cat owners [32–39]. This study aimed to assess the knowledge and practices (KP) of companion animal owners in Mainland Portugal concerning fleas, flea-borne pathogens, and related treatment and prevention measures.

## Methods

### Study population and sample size calculation

**Fig. 1:**
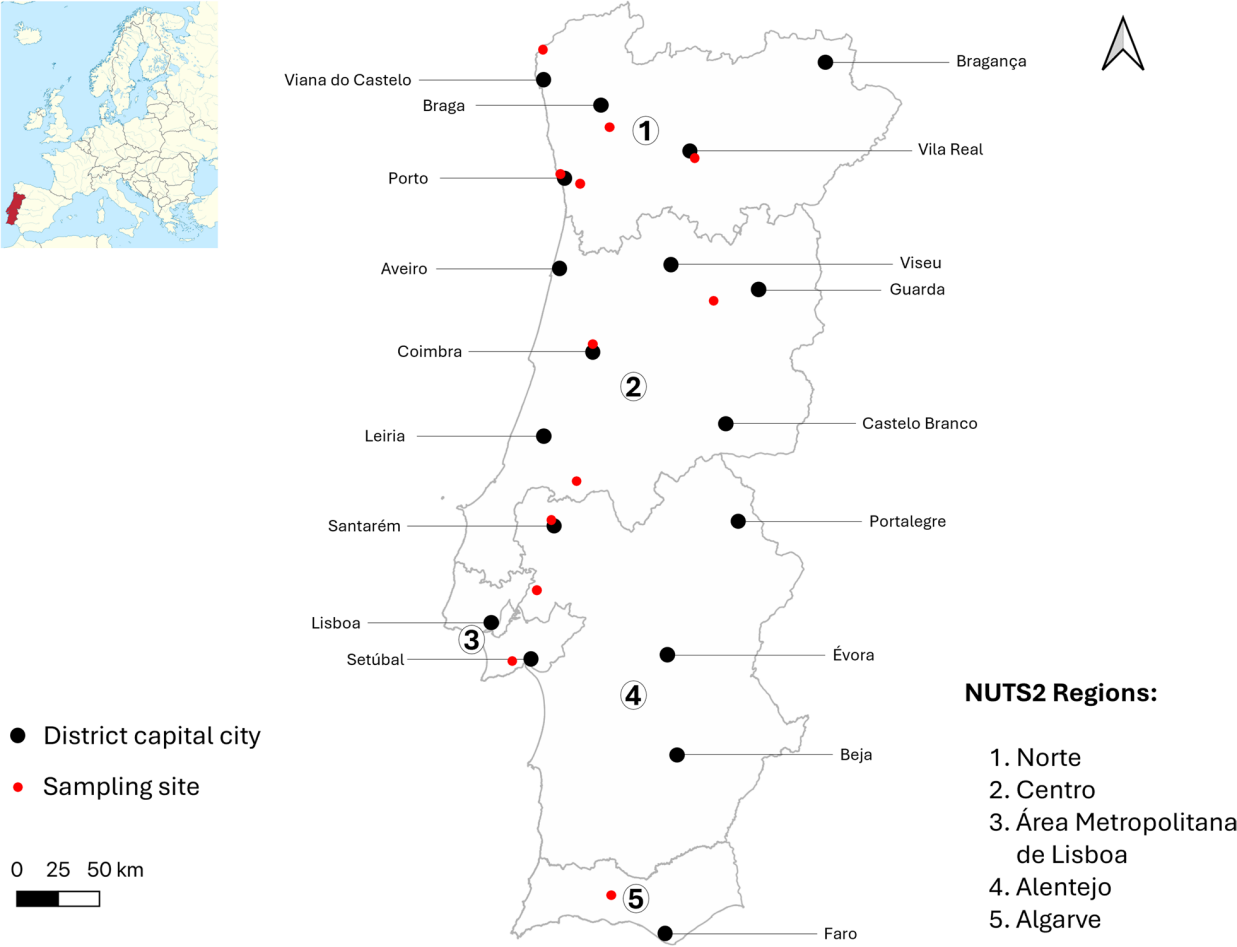
Geographical locations of the sampled sites (participating veterinary care centres) across the five Mainland Portuguese NUTS2 (Nomenclature of Territorial Units for Statistics) regions

From March 2022 to March 2023, a self-administered, structured, and anonymous questionnaire was distributed to companion animal owners attending veterinary care centres across the five Mainland Portuguese NUTS2 (Nomenclature of Territorial Units for Statistics) regions: Norte, Centro, Área Metropolitana de Lisboa (AML), Alentejo, and Algarve (Fig. 1). The inclusion criteria for participation were age of at least 18 years, owning a dog and/or cat at the time of the survey, and providing consent by signing the informed consent form.

Sample size was estimated using the Epitools© Epidemiological Calculators (https://epitools.ausvet.com.au). Since there were no previous similar studies, it was assumed that 50% of the population had poor knowledge of these topics and/or no adjusted practices. For a confidence level of 95% and a margin of error of 5%, the required sample size was calculated as 384 completed questionnaires. To allow for a 10% non-response rate, the adjusted sample size required was 427 participants.

### Data collection

The questionnaire aimed at collecting sociodemographic data and assessing animal owners’ knowledge of flea biology, flea-borne agents and associated diseases, and methods of prevention and control. Additionally, it aimed to understand the participants’ practices related to their pets’ control habits for these ectoparasites (Supplementary Fig. 1).

The questionnaire was constructed specifically for this study, although some questions were adapted from previous KP research on fleas and flea-borne pathogens. The questions were designed to address relevant knowledge topics and pet protective practices, while avoiding redundancy. Questions considered to be appropriate for the purpose of the project based on the consensus of all authors were included in the final version of the questionnaire. To improve ease/speed of completing the questionnaire, most questions were designed as multiple-choice; possible answers were selected by the authors to include all “correct” knowledge based on current scientific evidence and all the expected most common practices; additional plausible options were added to allow greater discrimination. Some multiple-choice questions permitted the selection of several options, which may result in total percentages exceeding 100% in the reported results.

The completion of the questionnaires by companion animal owners was voluntary and required participants to read an informational document about the study and sign an informed consent form. Participants had the option of completing the questionnaire either on paper or online (via Google Forms, accessible through a Quick Response [QR] code).

A pilot study was carried out with 35 individuals from different regions of the country, representing various age groups and education levels. Both the paper and online versions of the questionnaire were tested. The completion time was recorded, and participants’ hesitations, difficulties in understanding questions, and suggestions were considered to refine and prepare the final version of the questionnaire. Although the questionnaire was pilot-tested with a small group, formal reliability analysis (e.g., Cronbach’s alpha) was not performed, as this was beyond the scope of the current study.

### Data statistical analysis

Categorical variables extracted from the questionnaire were analysed mostly using the original categories provided as answer options, but regrouping was performed in some cases. Absolute and relative frequencies, as well as hypothesis testing and logistic regressions, were analysed using IBM^®^ SPSS^®^ Statistics version 29.0. Bar charts were created with Microsoft^®^ Excel^®^, and geographical representations were performed using QGIS^®^ version 3.22. Responses to each KP question were scored based on the criteria outlined in Supplementary Fig. 2. A total score for each individual was calculated for knowledge regarding fleas (Kf score), knowledge regarding flea-borne pathogens (Kp score), and practices (Pra score) by summing the individual scores for all questions in each category. The score ranges were as follows: Kf 0–4, Kp 0–4, Pra 0–7.

Descriptive statistics were reported as absolute frequencies and percentages for categorical variables, and as means with standard deviations or medians with interquartile ranges (IQRs) for continuous variables (e.g., age, Kf, Kp, and Pra scores). Group comparisons for categorical variables were conducted using the Pearson Chi-square test or Fisher’s exact test when the Chi-square test assumptions were not met. For continuous variables, the normality and homogeneity of variances= were assessed, and when assumptions were violated, the Mann–Whitney U test or Kruskal–Wallis test was applied for comparisons between two or more independent groups, respectively. A *P*-value of < 0.05 was considered statistically significant.

Recoding into binary variables was performed. Participants were divided into two groups based on the overall median score: equal to or above the median and below the median (Kf = 2.40, Kp = 1.85, Pra = 5.00). Multivariate analyses were conducted to identify sociodemographic factors associated with higher Kf, Kp, or Pra scores. These analyses utilised multiple binary logistic regression models, including variables that were statistically significant in univariate analyses (*P* < 0.05) and those deemed biologically relevant or potential confounders. The reference categories for independent variables are detailed in each multivariate analysis results table. For variables that remained significant, crude odds ratios (OR) were adjusted to produce adjusted odds ratios (aOR) with 95% confidence intervals (CI). The Hosmer–Lemeshow test was applied to evaluate the goodness of fit for each logistic regression model [40].

## Results

### Sociodemographic characteristics

A total of 550 participants were included in this study, of which 38.5% were exclusively dog owners, 28.7% were exclusively cat owners, and 32.7% owned both dog(s) and cat(s). Sociodemographic characteristics are summarised in Table 1. The median age was 40 years (IQR 28–52), and 69.9% were female. The percentage of female participants was significantly higher in “dog+cat” owners (Chi-square test, *χ*^2^ = 9.05, *df* = 2, *P* = 0.011). Concerning education level, 56.7% of participants reported having higher education and 30.2% secondary education. The most common NUTS2 region of residence was the AML (29.3%), followed by the Norte (25.2%), Alentejo (24.4%), Centro (18.5%), and Algarve (2.6%) regions; distribution by NUTS2 region was significantly different according to group (Chi-square test, *χ*^2^ = 21.84, *df* = 8, *P* = 0.005). Participants resided in 86 of the 278 municipalities of Mainland Portugal; the distribution of the number of participants by NUTS3 region is presented in Supplementary Fig. 3.

**Table 1:** Sociodemographic characteristics of the participants, overall and by group

	Total responses	Dog only	Cat only	Dog and cat	*P*-value
Total (*n*)	550	212	158	180	
Median age (years) (IQR)	40 (28–52)	40 (29–52.25)	36 (28–52)	42 (28–52.75)	0.780 (*H* = 0.496, *df* = 2)
Female sex (%)	69.9 (380/544)	64.4 (134/208)	66.9 (105/157)	78.8 (141/179)	0.011* (*χ*^2^ = 9.053, *df* = 2)
Education level ^a^ (%)					
Basic (1–4)	3.0	2.4	3.2	3.4	0.461 (*χ*^2^ = 5.669, *df* = 6)
	(16/540)	(5/205)	(5/157)	(6/178)	
Basic (5–9)	10.2	12.7	6.4	10.7	
	(55/540)	(26/205)	(10/157)	(19/178)	
Secondary (10–12)	30.2	29.8	34.4	27.0	
	(163/540)	(61/205)	(54/157)	(48/178)	
Higher (BS/MSc/PhD)	56.7	55.1	56.1	59.0	
	(306/540)	(113/205)	(88/157)	(105/178)	
Region of residence (%)					
Norte	25.2	21.3	33.5	22.5	0.005* (*χ*^2^ = 21.840, *df* = 8)
	(136/540)	(44/207)	(52/155)	(40/178)	
Centro	18.5	17.4	14.2	23.6	
	(100/540)	(36/207)	(22/155)	(42/178)	
AML	29.3	35.7	30.3	20.8	
	(158/540)	(74/207)	(47/155)	(37/178)	
Alentejo	24.4	23.2	20.0	29.8	
	(132/540)	(48/207)	(31/155)	(53/178)	
Algarve	2.6	2.4	1.9	3.4	
	(14/540)	(5/207)	(3/155)	(6/178)	
Unknown	1.8	2.4	1.9	1.1	
	(10/550)	(5/212)	(3/158)	(2/180)	

^a^Numbers in brackets refer to number of years of formal school education completed

*AML* Área Metropolitana de Lisboa; *IQR* interquartile range; *BS* bachelor of science; *MSc* master of science; *PhD* doctor of philosophy

*Statistically significant

The median number of pets (dogs or cats) owned by participants was 2 (IQR 1–3). The number of animals ranged from 0 to 12 for dogs and from 0 to 31 for cats. Ownership of a single dog or cat was mentioned by 55.1% of dog owners and 45.0% of cat owners, respectively. Living indoors and having access to/going outdoors (e.g., street, yard) was the pet lifestyle most often reported both for dogs (76.8%) and cats (48.6%). However, significantly different answers regarding lifestyle were noted between groups, with “dog+cat” owners more commonly reporting that their animal(s) lived exclusively outdoors and less commonly exclusively indoors compared with single-species owners, for both dogs (Chi-square test, *χ*^2^ = 12.91, *df* = 2, *P* = 0.002) and cats (Chi-square test, *χ*^2^ = 18.03, *df* = 2, *P* < 0.001).

### Knowledge about fleas

**Fig. 2:**
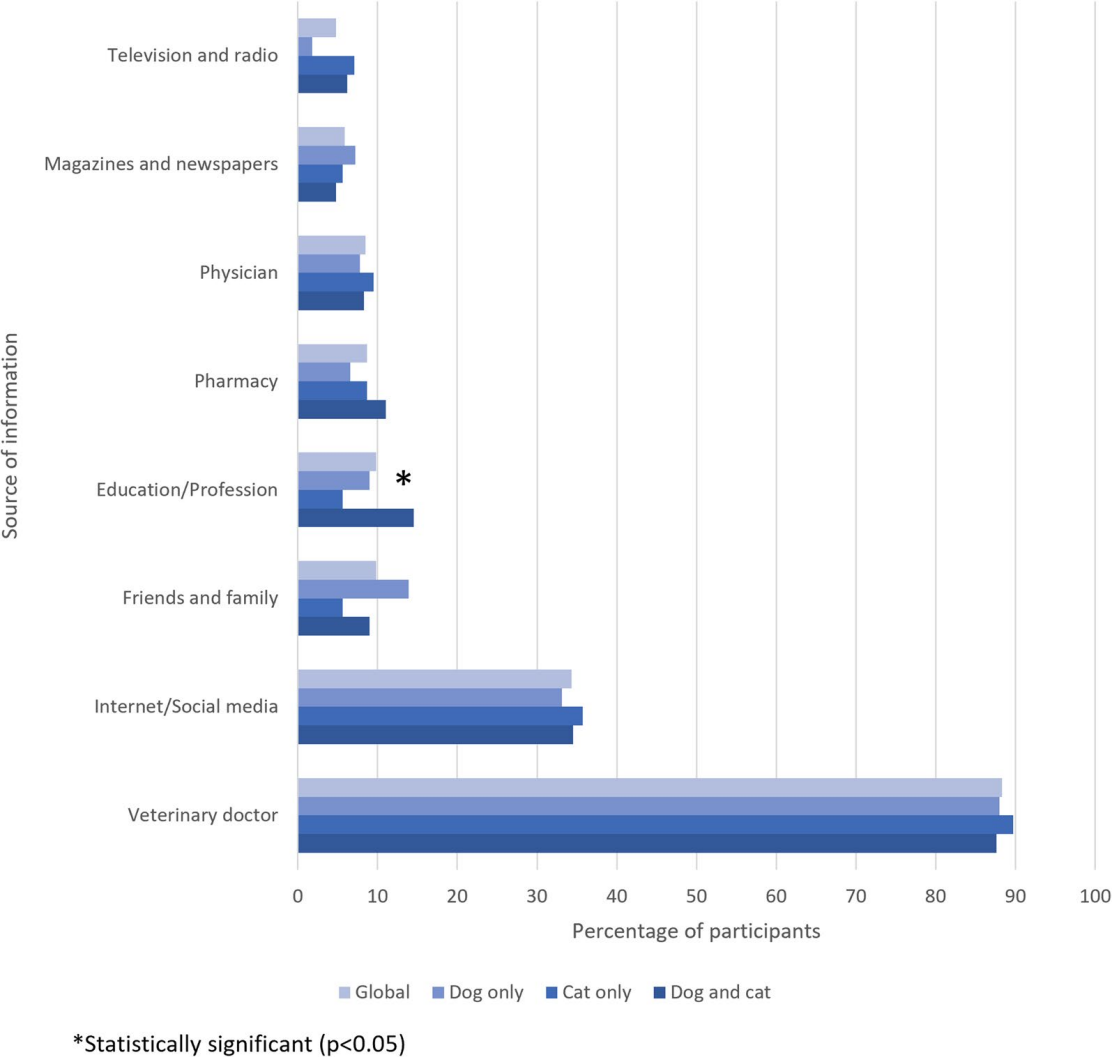
Percentage of participants, overall and by group, reporting different sources of information about fleas and flea-borne pathogens

Veterinary doctors were the most commonly reported sources of information about fleas and flea-borne pathogens (88.3%), followed by the internet and social media (34.3%). Less common sources, described by less than 10% of participants each, included friends and family, academic or educational activity, pharmacies, physicians, magazines and newspapers, and television and radio (Fig. 2). Academic or professional activity was the only source reported at a significantly different percentage between groups (more common in “dog+cat” owners) (Chi-square test, *χ*^2^ = 6.25, *df* = 2, *P* = 0.044).

**Fig. 3:**
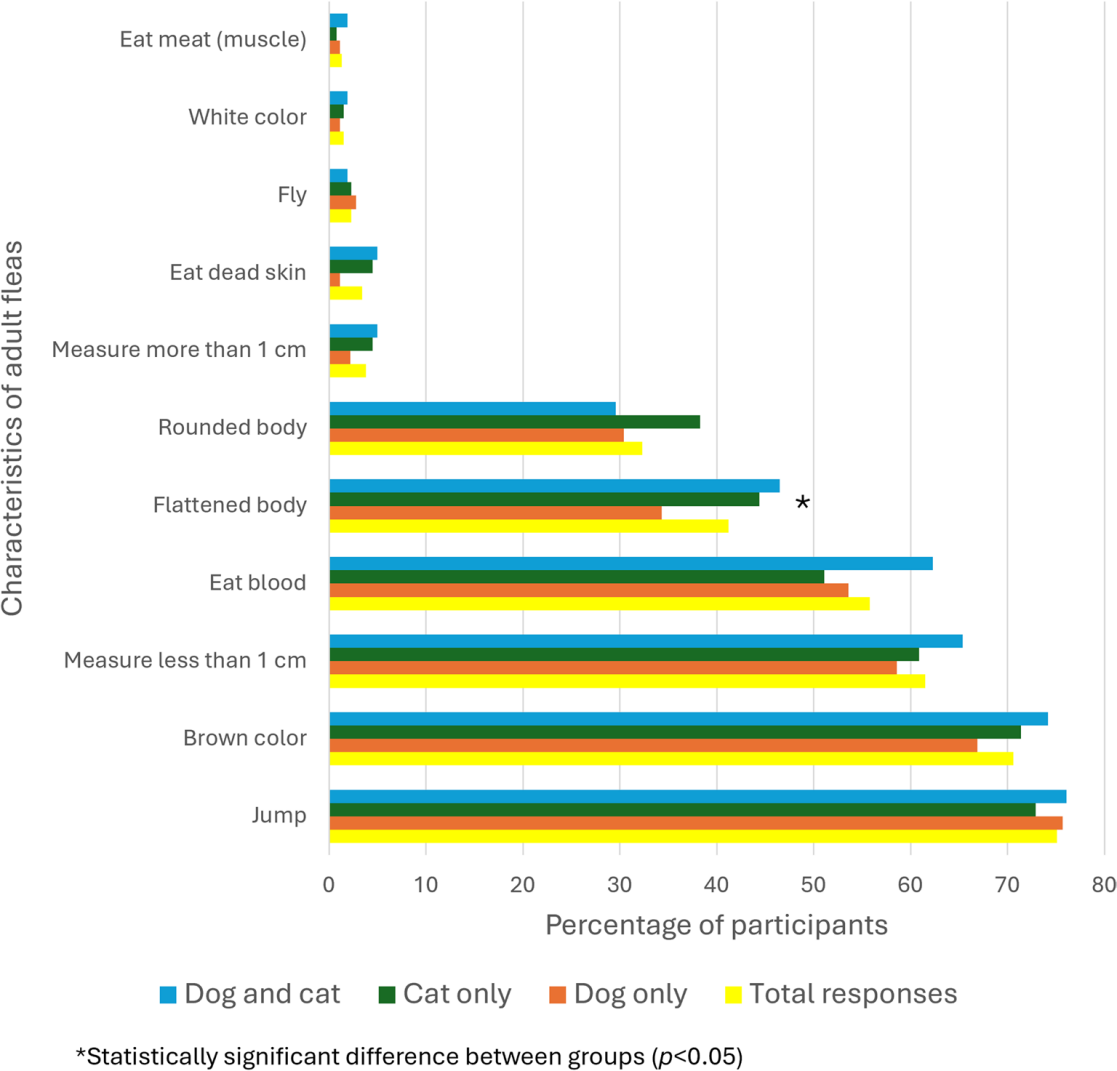
Selected characteristics of adult fleas, among the participants who recognised physical characteristics, overall and by group

Over 10% of participants were not able to recognise any characteristic of adult fleas. Among the participants who recognised physical characteristics, the most commonly selected were as follows: ability to jump (75.1%), brown colour (70.6%), measuring less than 1 cm (61.5%), eating blood (55.8%), a flattened body (41.2%), and a rounded body (32.3%). All the characteristics were selected with similar frequency among the three groups, except for flattened body (Chi-square test, *χ*^2^ = 6.02, *df* = 2, *P* = 0.049) (Fig. 3). When questioned about the most common way for animals to get fleas, 90.3% of participants mentioned that it was through the environment, outside the house (street, garden, park), 54.4% through another animal, 14.9% through the environment, at home (carpets, beds, floors), and 10.2% through people. “Cat only” owners significantly more often mentioned at home (Chi-square test, *χ*^2^ = 8.84, *df* = 2, *P* = 0.012) and through people (Chi-square test, *χ*^2^ = 30.23, *df* = 2, *P* < 0.001) as the most common way to get fleas (> 20% for each). Among participants who admitted knowing the period of activity of fleas (91.6%), most considered that they were active all year-round (58.2%) and 27.5% considered from spring to autumn (no significant difference between groups). A total of 13.9% of participants assumed not knowing whether the fleas that parasitise the dog/cat can also parasitise humans; of the remainder, most answered that fleas could also parasitise humans (87.6%) (Table 2).

**Table 2:** Answers to knowledge questions regarding fleas, overall and by group

	Total responses	Dog only	Cat only	Dog and cat	*P*-value
Most common way to get fleas (%)					
DK/CR	2.0	2.8	2.5	0.6	0.213
	(11/548)	(6/212)	(4/157)	(1/179)	(FET = 3.050)
Through the environment, outside	90.3	89.8	90.2	91.0	0.922
	(485/537)	(185/206)	(138/153)	(162/178)	(*χ*^2^ = 0.162, *df* = 2)
Through another animal	54.4	56.3	56.2	50.6	0.458
	(292/537)	(116/206)	(86/153)	(90/178)	(*χ*^2^ = 1.562, *df* = 2)
Through the environment, at home	14.9	9.7	20.9	15.7	0.012*
	(80/537)	(20/206)	(32/153)	(28/178)	(*χ*^2^ = 8.842, *df* = 2)
Through people	10.2	4.9	21.6	6.7	< 0.001*
	(55/537)	(10/206)	(33/153)	(12/178)	(*χ*^2^ = 30.228, *df* = 2)
Other^a^	0.2	0.5	0.0	0.0	NA
	(1/537)	(1/206)	(0/153)	(0/178)	
Period of activity (%)					
DK/CR	8.4	10.4	9.6	5.0	0.128
	(46/548)	(22/211)	(15/157)	(9/180)	(*χ*^2^ = 4.105, *df* = 2)
All year-round	58.2	58.2	57.7	58.5	0.535 (*χ*^2^ = 5.068, *df* = 6)
	(292/502)	(110/189)	(82/142)	(100/171)	
From spring to autumn	27.5	24.9	32.4	26.3	
	(138/502)	(47/189)	(46/142)	(45/171)	
Only summer	7.6	9.5	4.9	7.6	
	(38/502)	(18/189)	(7/142)	(13/171)	
Other^b^	6.8	7.4	4.9	7.6	
	(34/502)	(14/189)	(7/142)	(13/171)	
Parasitisation of humans (%)					
DK/CR	13.9	15.2	15.3	11.2	0.446
	(76/545)	(32/210)	(24/157)	(20/178)	(*χ*^2^ = 1.617, *df* = 2)
Yes	87.6	88.8	85.0	88.6	1.224
	(411/469)	(158/178)	(113/133)	(140/158)	(*χ*^2^ = 1.617, *df* = 2)

^a^Veterinary clinics (*n* = 1)

^b^Spring and summer (*n* = 13); only spring (*n* = 17); only winter (*n* = 4)

Abbreviations: *DK/CR* don’t know/can’t remember

*Statistically significant

### Knowledge about infections transmitted by fleas

Transmission of pathogens by fleas was recognised by 81.9% of participants (Table 3). The flea’s bite was the most frequently selected route of transmission to animals (96.3%) and humans (84.3%), followed by direct contact with an animal for humans (17.3%) and flea’s faeces for both animals (14.8%) and humans (11.5%). Ingestion of fleas was rarely identified as a possible route of transmission to humans (4.7%). Animal scratch was the only route of transmission selected at a significantly different frequency between groups—more commonly in “cat only” owners (Fisher’s exact test, FET = 8.60, *P* = 0.023). Only 12.7% of participants who knew that fleas transmitted pathogens could name specific agents; the most common in these cases were *Yersinia pestis* (42.1%) and *Rickettsia typhi* (31.6%). Although *D. caninum* was more commonly mentioned by “dog only” or “dog+cat” owners and *Mycoplasma* sp. and *Bartonella* sp. were more commonly mentioned by “cat only” owners, these differences did not reach statistical significance. Some participants (3.8%), while not recognising specific pathogens, were knowledgeable about the general group (such as “bacteria”, “intestinal parasites”, or “internal parasites”), while others (8.3%) simply recognised signs attributable to flea-borne infections (such as “fever” or “anaemia”). Additionally, 20.1% of participants mentioned cutaneous manifestations directly related to the flea’s bite (such as “allergy” or “dermatitis”).

**Table 3:** Answers to knowledge questions regarding flea-borne pathogens, overall and by group

	Total responses	Dog only	Cat only	Dog and cat	*P*-value
Transmission of pathogens (%)					
Yes	81.9	80.6	82.2	83.2	0.788
	(448/547)	(170/211)	(129/157)	(149/179)	(*χ*^2^ = 0.477, *df* = 2)
Routes of transmission to animals (%)					
DK/CR	3.3	3.5	2.3	4.0	0.758
	(15/448)	(6/170)	(3/129)	(6/149)	(FET = 0.646)
Flea’s bite	96.3	94.5	97.6	97.2	0.346
	(417/433)	(155/164)	(123/126)	(139/143)	(FET = 2.416)
Flea’s faeces	14.8	11.6	17.5	16.1	0.326
	(64/433)	(19/164)	(22/126)	(23/143)	(*χ*^2^ = 2.240, *df* = 2)
Ingestion of flea	9.0	7.9	10.3	9.1	0.779
	(39/433)	(13/164)	(13/126)	(13/143)	(*χ*^2^ = 0.499, *df* = 2)
Routes of transmission to humans (%)					
DK/CR	4.7	4.7	7.8	2.0	0.078
	(21/448)	(8/170)	(10/129)	(3/149)	(*χ*^2^ = 5.096, *df* = 2)
Flea’s bite	84.3	83.3	79.8	89.0	0.111
	(360/427)	(135/162)	(95/119)	(130/146)	(*χ*^2^ = 4.391, *df* = 2)
Contact with animal	17.3	19.1	17.6	15.1	0.638
	(74/427)	(31/162)	(21/119)	(22/146)	(*χ*^2^ = 0.898, *df* = 2)
Flea’s faeces	11.5	8.6	12.6	13.7	0.343
	(49/427)	(14/162)	(15/119)	(20/146)	(*χ*^2^ = 2.140, *df* = 2)
Animal bite	8.0	6.2	9.2	8.9	0.562
	(34/427)	(10/162)	(11/119)	(13/146)	(*χ*^2^ = 1.151, *df* = 2)
Ingestion of flea	4.7	4.9	5.0	4.1	0.921
	(20/427)	(8/162)	(6/119)	(6/146)	(*χ*^2^ = 0.166, *df* = 2)
Animal scratch	4.0	1.9	8.4	2.7	0.023*
	(17/427)	(3/162)	(10/119)	(4/146)	(FET = 8.598)
Infectious agents transmitted (%)					
DK/CR/Non-flea associated	87.3	87.6	88.4	85.9	0.814
	(391/448)	(149/170)	(114/129)	(128/149)	(*χ*^2^ = 0.412, *df* = 2)
* Yersinia pestis*	42.1	38.1	46.7	42.9	0.873
	(24/57)	(8/21)	(7/15)	(9/21)	(*χ*^2^ = 0.271, *df* = 2)
* Rickettsia typhi*	31.6	33.3	33.3	28.6	0.933
	(18/57)	(7/21)	(5/15)	(6/21)	(*χ*^2^ = 0.139, *df* = 2)
* Dipylidium caninum*	26.3	23.8	13.3	38.1	0.266
	(15/57)	(5/21)	(2/15)	(8/21)	(FET = 2.875)
* Mycoplasma* sp.	21.1	14.3	40.0	14.3	0.117
	(12/57)	(3/21)	(6/15)	(3/21)	(FET = 4.397)
* Bartonella* sp.	8.8	0	20.0	9.5	0.113
	(5/57)	(0/21)	(3/15)	(2/21)	(FET = 2.193)

*DK/CR* don’t know/can’t remember

*Statistically significant

### Practices related to fleas

Prior detection of fleas in pets was recognised by 63.1% of participants, with a significantly higher proportion in “dog+cat” owners (Chi-square test, *χ*^2^ = 29.27, *df* = 2, *P* < 0.001). The most commonly reported methods for detection of fleas were by the observation of fleas on the animal (64.8%), especially for cat owners (Chi-square test, *χ*^2^ = 7.35, *df* = 2, *P* = 0.025), and through the animal’s behaviour (e.g. scratching more than usual) (63.4%), especially for dog owners (Chi-square test, *χ*^2^ = 6.60, *df* = 2, *P* = 0.037). Treatment against fleas was assumed to be practised by 94.3% of participants in general, albeit in a significantly lower proportion of “cat only” owners (Chi-square test, *χ*^2^ = 20.56, *df* = 2, *P* < 0.001). Common reasons given for flea treatment included to prevent getting fleas (88.6%), because the veterinary doctor recommended it (23.8%), and as a treatment when fleas were detected (18.6%). Reasons for not performing flea treatment in pets (*n* = 21) included absence of fleas in the pet(s) (66.7%), perception of low risk for fleas and flea-borne pathogens (23.8%), complicated treatment scheme for animal does not allow application (14.3%), no recommendation by the veterinary doctor (9.5%), and high cost (4.8%). Among participants who knew the frequency of flea treatment of their pet(s), most reported every 3 to 4 months (46.2%). The most commonly reported products used for flea treatment were spot-on (64.7%), followed by chewable or hard tablets (41.6%) and collar (30.1%). Significant differences were seen among groups in the proportion of use of these products, with spot-on more frequently reported among cat owners (Chi-square test, *χ*^2^ = 36.47, *df* = 2, *P* < 0.001), while tablets and collars were more frequently reported among dog owners (Chi-square test, *χ*^2^ = 14.87, *df* = 2, *P* = 0.001 and Chi-square test, *χ*^2^ = 48.78, *df* = 2, *P* < 0.001, respectively). Among participants owning multiple animals, 90.1% mentioned treating all the animals when one of them had fleas, although this proportion was significantly lower for “dog+cat” owners (Chi-square test, *χ*^2^ = 8.73, *df* = 2, *P* = 0.013). In “dog+cat” owners, 64.8% said they treated both dogs and cats with the same frequency and 27.8% with different frequency. Reasons for lack of treatment cited by the remaining 7.4% who did not treat both species of companion animals (*n* = 13) were as follows: the cat(s) did not go outside (46.2%), only the dog(s) had fleas (30.8%), it was too expensive (15.4%), and some animals did not allow their owners to apply the treatment (7.8%). Concerning measures to control infestation, the most frequently selected were cleaning and vacuuming the house/animal’s resting places (79.3%), asking the veterinarian for advice on flea prevention and control (63.6%), brushing the animal with a comb indicated for the removal of fleas (45.8%), and applying insecticidal products in the animal’s home/resting places (44.3%). Of these, the first two were significantly more frequently selected by “cat only” owners (Chi-square test, *χ*^2^ = 7.30, *df* = 2, *P* = 0.026 and Chi-square test, *χ*^2^ = 11.17, *df* = 2, *P* = 0.004, respectively), while for the other two there were no significant differences among groups (Table 4).

**Table 4:** Answers to practices questions, overall and by group

	Total responses	Dog only	Cat only	Dog and cat	*P*-value
Detection of fleas in pet (%)					
Yes	63.1	57.1	53.2	78.9	< 0.001*
	(347/550)	(121/212)	(84/158)	(142/180)	(*χ*^2^ = 29.272, *df* = 2)
Method of detection (%)					
Observation of fleas on animal	64.8	55.4	69.0	70.4	0.025*
	(225/347)	(67/121)	(58/84)	(100/142)	(*χ*^2^ = 7.352, *df* = 2)
Animal’s behaviour	63.4	71.1	53.6	62.7	0.037*
	(220/347)	(86/121)	(45/84)	(89/142)	(*χ*^2^ = 6.600, *df* = 2)
Bites on members of household	16.7	12.4	17.9	19.7	0.270
	(58/347)	(15/121)	(15/84)	(28/142)	(*χ*^2^ = 2.620, *df* = 2)
The veterinary doctor said	10.4	8.3	14.3	9.9	0.368
	(36/347)	(10/121)	(12/84)	(14/142)	(*χ*^2^ = 2.002, *df* = 2)
Observation of fleas in the environment	10.1	9.1	13.1	9.2	0.575
	(35/347)	(11/121)	(11/84)	(13/142)	(*χ*^2^ = 1.107, *df* = 2)
Signs of allergic reaction	9.2	8.3	8.3	10.6	0.772
	(32/347)	(10/121)	(7/84)	(15/142)	(*χ*^2^ = 0.517, *df* = 2)
Treatment against fleas (%)					
Yes	94.3	97.6	87.3	96.7	< 0.001*
	(517/548)	(205/210)	(138/158)	(174/180)	(*χ*^2^ = 20.556, *df* = 2)
Reason for treatment (%)					
Prevent from getting fleas	88.6	91.2	85.5	87.9	0.250
	(458/517)	(187/205)	(118/138)	(153/174)	(*χ*^2^ = 2.774, *df* = 2)
Recommendation by veterinarian	23.8	26.3	24.6	20.1	0.352
	(123/517)	(54/205)	(34/138)	(35/174)	(*χ*^2^ = 2.087, *df*f = 2)
Treatment of fleas	18.6	11.7	20.3	25.3	0.003*
	(96/517)	(24/205)	(28/138)	(44/174)	(*χ*^2^ = 11.848, *df* = 2)
Heard on TV/radio/social media	1.2	1.0	2.2	0.6	0.453
	(6/517)	(2/205)	(3/138)	(1/174)	(FET = 1.708)
Frequency of treatment (%)					
DK/CR	7.0	9.8	5.1	5.2	0.132
	(36/517)	(20/205)	(7/137)	(9/174)	(*χ*^2^ = 4.049, *df* = 2)
Once a month	22.2	22.7	21.5	22.4	0.969
	(107/481)	(42/185)	(28/130)	(37/165)	(*χ*^2^ = 0.062, *df* = 2)
Every 3–4 months	46.2	43.8	43.8	50.9	0.334
	(222/481)	(81/185)	(57/130)	(84/165)	(*χ*^2^ = 2.196, *df* = 2)
Every 6–8 months	23.7	29.7	16.9	22.4	0.028*
	(114/481)	(55/185)	(22/130)	(37/165)	(*χ*^2^ = 7.159, *df* = 2)
Once a year	8.5	5.4	16.9	5.5	< 0.001*
	(41/481)	(10/185)	(22/130)	(9/165)	(*χ*^2^ = 16.032, *df* = 2)
Only when fleas are detected	3.1	2.7	3.1	3.6	0.881
	(15/481)	(5/185)	(4/130)	(6/165)	(*χ*^2^ = 0.252, *df* = 2)
Products for treatment (%)					
Pipettes (spot-on)	64.7	49.8	80.1	70.1	< 0.001*
	(333/515)	(102/205)	(109/136)	(122/174)	(*χ*^2^ = 36.468, *df* = 2)
Chewable or hard tablets	41.6	44.4	27.9	48.9	0.001*
	(214/515)	(91/205)	(38/136)	(85/174)	(*χ*^2^ = 14.870, *df* = 2)
Collar	30.1	43.4	8.1	31.6	< 0.001*
	(155/515)	(89/205)	(11/136)	(55/174)	(*χ*^2^ = 48.783, *df* = 2)
Shampoo	3.3	5.4	0.7	2.9	0.052
	(17/515)	(11/205)	(1/136)	(5/174)	(FET = 5.599)
Spray	1.9	1.5	1.5	2.9	0.611
	(10/515)	(3/205)	(2/136)	(5/174)	(FET = 1.147)
Treating all animals (%)					
Yes	90.1	93.6	97.1	85.4	0.013*
	(273/303)	(73/78)	(66/68)	(134/157)	(*χ*^2^ = 8.726, *df* = 2)
Measures to control infestation (%)					
Clean and vacuum the house/animal’s resting places	79.3	73.3	83.7	82.4	0.026*
	(424/535)	(151/206)	(128/153)	(145/176)	(*χ*^2^ = 7.296, *df* = 2)
Ask the veterinarian for advice on flea prevention and control	63.6	59.7	74.5	58.5	0.004*
	(340/535)	(123/206)	(114/153)	(103/176)	(*χ*^2^ = 11.166, *df* = 2)
Brush the animal with a comb indicated for the removal of fleas	45.8	45.6	45.8	46.0	0.997
	(245/535)	(94/206)	(70/153)	(81/176)	(*χ*^2^ = 0.006, *df* = 2)
Apply insecticidal products in the animal’s home/resting places	44.3	43.2	42.5	47.2	0.641
	(237/535)	(89/206)	(65/153)	(83/176)	(*χ*^2^ = 0.888, *df* = 2)
Clean and vacuum the car	19.8	22.8	15.7	19.9	0.245
	(106/535)	(47/206)	(24/153)	(35/176)	(*χ*^2^ = 2.810, *df* = 2)
Keep the animal indoors from sunset until sunrise	5.0	1.5	9.8	5.1	0.002*
	(27/535)	(3/206)	(15/153)	(9/176)	(*χ*^2^ = 12.769, *df* = 2)
Use safety nets on windows and doors	4.1	3.9	5.9	2.8	0.374
	(22/535)	(8/206)	(9/153)	(5/176)	(*χ*^2^ = 1.965, *df* = 2)

*DK/CR* don’t know/can’t remember; **NA** not applicable; TV television

*Statistically significant

### Scoring KP and associations with sociodemographic factors

The distribution of individual scores for knowledge regarding fleas (Kf), knowledge regarding flea-borne pathogens (Kp), and practices (Pra) is represented in Supplementary Fig. 4. Median Kf, Kp, and Pra scores were not significantly different between the groups of “dog only”, “cat only”, and “dog+cat” owners, as represented in Table 5.

**Table 5:** Median scores for knowledge regarding fleas, knowledge regarding flea-borne pathogens, and practices, overall and by group

	Total responses	Dog only	Cat only	Dog and cat	*P*-value
Total (*n*)	550	212	158	180	
K score (fleas), median (IQR)	2.40 (1.85–2.90)	2.40 (1.65–2.89)	2.40 (1.80–2.85)	2.60 (1.90–2.94)	0.063 (*H* = 5.536, *df* = 2)
K score (pathogens), median (IQR)	1.85 (1.25–1.85)	1.85 (1.25–1.85)	1.85 (1.25–1.85)	1.85 (1.25–1.85)	0.365 (*H* = 2.017, *df* = 2)
Pra score, median (IQR)	5.00 (4.00–5.00)	5.00 (4.00–6.00)	5.00 (4.00–5.00)	4.50 (3.50–5.00)	0.085 (*H* = 4.937, *df* = 2)

*K* knowledge; *Pra* practices; *IQR* interquartile range

*Statistically significant

Univariate analysis of sociodemographic variables associated with Kf, Kp, or Pra scores equal to or above median is listed in Supplementary Table 1. Factors associated with a Kf score equal to or above the median (2.40) in univariate analysis were younger age (especially ≤ 50 years), higher level of education, and residing in the NUTS2 Centro, AML or Algarve regions. In multivariate analysis, all these factors remained statistically significant. Factors associated with a Kp score equal to or above the median (1.85) in univariate analysis were female sex, younger age (especially ≤ 40 years old), higher level of education, and residing in the NUTS2 Centro, AML, or Algarve regions. In multivariate analysis, all these factors remained statistically significant, except for age ≤ 40 years. Lastly, factors associated with a Pra score equal to or above median (5.0) in univariate analysis were higher level of education, residing in the NUTS2 Centro or AML region, being a “dog” or “cat” owner, having a Kf score ≥ 2.40, and having a Kp score ≥ 1.85. In multivariate analysis, however, only residing in the NUTS2 Centro or AML regions or being a “dog” or “cat” owner remained statistically significant (Table 6).

**Table 6:** Potential factors for scores equal to or above median for (a) knowledge regarding fleas, (b) knowledge regarding flea-borne pathogens, and (c) practices, according to logistic regression models to estimate crude and adjusted odds ratio values

(a)	Potential risk factor	Univariate			Multivariate			
		% in Sample	Crude OR	95% CI	Adjusted OR		95% CI	*P*-value
Kf ≥ 2.40	Female sex	70.5	1.34	0.92–1.94	1.13		0.76–1.68	0.556
	Age ≤ 50 years	72.0	2.12	1.44–3.10	1.84		1.23–2.74	0.003*
	Higher education^a^	56.7	2.40	1.70–3.41	2.05		1.42–2.96	< 0.001*
	AML/Centro/Algarve	50.4	1.85	1.31–2.60	1.54		1.07–2.21	0.021*
	Dog+cat owner	32.7	1.36	0.95–1.94	1.28		0.87–1.88	0.212
	Constant				0.354			< 0.001*
	Hosmer–Lemeshow test				Sig. = 0.311			

b)	Potential risk factor	Univariate			Multivariate			
		% in Sample	Crude OR	95% CI	Adjusted OR		95% CI	*P*-value
Kp ≥ 1.85	Female sex	70.5	1.74	1.19–2.53	1.62		1.07–2.45	0.023*
	Age ≤ 40 years	50.4	1.71	1.20–2.43	1.39		0.94–2.04	0.098
	Higher education^a^	56.7	2.44	1.70–3.48	2.03		1.37–3.01	< 0.001*
	AML/Centro/Algarve	50.4	2.00	1.40–2.85	1.83		1.23–2.73	0.003*
	Multiple pets	63.0	1.36	0.94–1.98	1.38		0.92–2.07	0.118
	Constant				0.432			0.001
	Hosmer–Lemeshow test				Sig. = 0.774			

c)	Potential risk factor	Univariate			Multivariate			
		% in Sample	Crude OR	95% CI	Adjusted OR		95% CI	*P*-value
Pra ≥ 5	Age ≤ 60 years	88.8	2.39	1.36–4.20	1.89		1.01–3.53	0.047*
	Higher education^a^	56.7	1.67	1.18–2.35	1.21		0.82–1.78	0.336
	AML/Centro	47.8	1.75	1.24–2.46	1.54		1.05–2.24	0.026*
	Only cat or only dog owner	67.3	1.76	1.23–2.52	1.81		1.14–2.87	0.011*
	Single pet	63.0	1.25	0.87–1.79	1.06		0.68–1.67	0.793
	Kf ≥ 2.40	53.8	1.46	1.04–2.04	1.23		0.84–1.79	0.290
	Kp ≥ 1.85	61.6	1.68	1.19–2.38	1.46		0.99–2.15	0.059
	Constant				0.384			0.007
	Hosmer–Lemeshow test				Sig. = 0.791			

^a^Completed bachelor’s degree or above

*Kf* knowledge (fleas); *Kp* knowledge (pathogens); *Pra* practices; *AML* Área Metropolitana de Lisboa; *OR* odds ratio; *CI* confidence interval

*Statistically significant

## Discussion

Fleas are among the most important worldwide ectoparasites of dogs and cats. Besides being responsible for causing direct deleterious effects such as anaemia and dermatological problems, they are also responsible for the transmission of numerous pathogens, some of which are zoonotic [30]. In Portugal, flea-borne infections have been reported in both companion animals and humans [10–28], highlighting the importance of public education regarding the measures to be taken to reduce the risk of exposure of dogs, cats, and humans to these ectoparasites and the pathogens they transmit. In the present study, a total of 550 companion animal owners in Mainland Portugal responded to a questionnaire regarding fleas, flea-borne pathogens, and their management: 38.5% were dog owners only, 28.7% were cat owners only, and 32.7% owned both animal species. Dog owners usually represent the largest percentage of participants in surveys conducted in the country [33, 34, 39], a trend that might be related to the fact that dogs are the most common pets in Portugal [9] and globally have greater access to veterinary care [41], which increases the frequency of interactions between dog owners and surveys.

Women made up 69.9% of the participants, which aligns with findings from previous studies on pet ownership, zoonoses, and parasite control awareness [35–39, 42]. This trend is likely related to a higher level of concern among women for the health and well-being of their pets. The mean age of pet owners who responded to the questionnaire was 40 years, and the majority had a secondary or higher level of education, which is similar to what was observed in other studies [35, 36, 38, 39]; this trend may reflect a greater interest among middle-aged adults and individuals with higher educational qualifications to participate in this kind of KP research studies. Geographically, the majority of participants were based in the Área Metropolitana de Lisboa (AML), whereas Algarve had the lowest response rate. Although the questionnaire was distributed through several veterinary clinics across Mainland Portugal, the number of participating clinics in the Algarve was relatively low, potentially influencing respondent numbers. This distribution pattern is consistent with previous national studies [34], [42] and may reflect regional variations in interest levels or cultural differences in pet care practices and perceptions.

Few studies have assessed companion animal owners’ level of knowledge regarding fleas and flea-borne pathogens [32, 35]. In a study from Hungary [32], most dog and cat owners were unaware that fleas can harm both animals and humans or that the environment can be a source of infestation, while in a study from Malaysia [35] about 40% of cat owners recognised fleas as pathogen vectors and were aware that infestations can originate from the environment. In addition, more than half of the Malaysian cat owners were also able to identify the size, food source, and movement of the fleas, which could be because most of the responders had previously experienced flea infestations in cats. In this study, over 70% of participants correctly identified the flea’s body colour and movement, while more than half recognised its size and diet, demonstrating a reasonable understanding of flea biology. Companion animal owners also recognised the environment, mainly outdoor spaces, and contact with infested animals as common sources of flea infestations, as well as the fact that fleas may remain active year-round, reflecting their awareness of the flea life cycle. Interestingly, cat-only owners were significantly more aware than the other two owner groups that fleas have a flat body shape, and that indoor environments and humans can also be a source of infestation. In contrast to previous studies [32, 35], most participants recognised fleas as vectors of pathogens affecting both animals and humans, with bites being the most commonly known transmission route. However, as in previous studies, only a few could name the specific etiological agents. Knowledge about pathogen transmission through scratches was significantly higher among cat-only owners. Although the identification of pathogens did not differ among the three groups, cat-only owners most frequently mentioned the etiological agents of cat-scratch disease and mycoplasmosis, while *D. caninum* was more frequently noted by dog-only and dog+cat owners, suggesting that some participants were able to correctly associate certain pathogens with the animal species they affect.

In this study, multivariate analysis confirmed trends observed in previous CPP questionnaires regarding sex, age, and education level. Specifically, a higher awareness of fleas and flea-borne pathogens was associated with participants who had higher education, were under 50 years of age (Kf only), and were female (Kp only). Knowledge was also higher among participants living in the AML, Centro, and Algarve regions. Interestingly, despite the low number of participants from the Algarve region, their greater knowledge may be linked to the participation of individuals particularly concerned about the seriousness of these ectoparasites.

The considerable ability of participants to recognise fleas, to associate flea presence with changes in animals’ behaviour, and to understand the role of these ectoparasites in pathogen transmission may be attributed to information provided by veterinarians or previous experiences with flea infestations in their companion animals. In fact, about two-thirds of the participants reported prior flea infestations, which may have prompted them to seek advice from veterinarians, the most common reported source of information on fleas and flea-borne pathogens, followed by social media and the internet. The acquisition of information about zoonotic infections from veterinarians emphasises their important role in raising the awareness of owners about the ways of transmission of the pathogens to their pets and themselves [35], although in some cases the proactivity of veterinarians in passing on knowledge has been considered negligible [36, 39]. Routine consultations provide an important opportunity for veterinarians to educate owners about the health risks fleas pose. To enhance the impact of these interactions, clear and simple educational materials, such as brochures, posters, and infographics, can be displayed in clinic waiting areas to reinforce key messages and support owner understanding. Curiously, and despite the fact that advertisements on TV about the application of ectoparasiticides to prevent arthropods and vector-borne infections was a common source of information identified in previous studies [36, 43], in the present study very few participants reported the use of this communication channel to obtain information about fleas and flea-borne pathogens. Similarly, the low demand for information on these topics from physicians (less than 10% of participants) suggests that healthcare professionals are not perceived as key sources of education on the risks of vector-borne zoonotic agents for pet-owning patients [44].

To effectively avoid and eliminate fleas, prevent flea-borne infections, and manage flea allergy dermatitis, an integrated control strategy should target both immature and adult flea stages. This strategy may involve using products containing insect growth regulators or juvenile hormone analogues, formulations with repellent or fast-killing properties, or those with combined effects on both the animal and its environment. To prevent and eliminate infestations, a range of products are available in various formulations, including collars, spot-on treatments, sprays, powders, shampoos, chewable or hard tablets, and injectables [30, 45].

ESCCAP recommends year-round flea prevention to cover the complete activity period of the ectoparasite, as exposure is difficult to avoid [31].

Portugal has a temperate Mediterranean climate [46], which likely creates favourable conditions for flea populations to persist throughout the year. This climate, characterized by mild winters and warm to hot summers, combined with the common outdoor access granted to pets, increases the risk of flea infestation and underscores the need for continuous preventive measures. Most participants stated that their companion animals were treated against fleas as a preventive measure, aligning with previous studies on ectoparasite control practices among dog and cat owners [33, 34, 39, 47]. About half of the participants treated their pets for ectoparasites every 3 to 4 months, followed by every 6 to 8 months, and once per month. The preference for spot-on treatments among cat owners aligns with other studies, likely due to their ease of application [47]. Similarly, oral tablets were less commonly referred to as being used by this group, probably because cats are considered to be difficult to medicate orally [47]. As the questionnaire did not include the active ingredient of the products used, the accuracy of their application according to the manufacturer’s recommendations cannot be assessed. Given that spot-on treatments were the most commonly used route among cat-only owners and owners of both animal species, and with few exceptions are effective for only 3–4 weeks, it is likely that many pets were not treated at the correct frequency. The same conclusion can be drawn for the administration of ectoparasites in tablet form, the second most commonly used product by dog owners and owners of both animal species, since the treatment schedule is between 4 and 12 weeks. Reasons for non-compliance may relate to a lack of awareness about the importance of continuous prevention, the absence of infestation (thus reducing adherence to long-term prevention protocols), or financial constraints, with the latter reported by 15.4% of participants. Flea treatment rates for all animals in the household were significantly lower among dog and cat owners than among those who owned only dogs or only cats, although the overall frequency of flea treatment did not differ significantly between these groups. A survey of dog owners in Thailand found that the cost of flea and tick prevention, especially in households with multiple dogs, was linked to less frequent use of preventive treatments. Most owners tended to use products only when their dogs were infested [37]. In the present study, only a small percentage of participants claimed to treat their animals only when fleas were detected.

Effective prophylactic treatment should be used in conjunction with environmental control to target all life stages. Mechanical environmental measures such as frequent vacuuming, washing pet bedding, and cleaning all areas that may harbour eggs can significantly reduce the household flea burden [6, 32, 50]. Interestingly, cleaning, vacuuming, and applying insecticides to the house and the animal’s resting places, along with brushing the animal, were the most frequently mentioned measures for controlling infestation, suggesting that many owners are aware of the importance of these practices in managing flea infestations. Cat-only owners were significantly more likely to choose the practice of cleaning areas with a possible higher risk of infestation and presented greater willingness to ask veterinarians for advice on preventing and controlling fleas. This, together with their greater knowledge of flea morphology and the fact that indoor environments and humans can also be sources of infestation, indicates a previous history of contact with fleas. In fact, 53.2% of the respondents of this group reported prior experience with fleas, which likely explains a greater motivation to apply preventative measures. Interestingly, a significantly higher proportion of participants in this group reported keeping their animals indoors from sunset to sunrise as a control measure. This may reflect confusion with other insects active during that time, such as mosquitoes and phlebotomine sand flies.

As mentioned earlier, multivariate analysis indicated that higher education was associated with greater knowledge of fleas and flea-borne pathogens. However, this knowledge did not lead to a higher adoption of protective practices. This discrepancy could be related to the perception that general protective measures against arthropods and arthropod-borne infections are also effective against fleas, suggesting that specific flea control measures may be unnecessary.

Conversely, higher practice scores were observed in owners under 60 years old, those living in the AML or Centro regions, and those owning only cats or only dogs. This may reflect a greater concern for preventive measures among middle-aged participants and individuals with higher knowledge of fleas and flea-borne pathogens. Additionally, owning a single species might lead to more focused and effective infestation prevention strategies.

Although over 30% of Portuguese households owned at least one dog or cat in 2022, respondents in this study came from only 86 of the 278 municipalities in Mainland Portugal. This limited geographical representation may have hindered a detailed analysis of how sociodemographic differences influence knowledge about fleas, the diseases they can cause, and management measures for these ectoparasites. As a result, the study’s ability to accurately identify trends in pet owners’ KP may have been affected. Future research should address this limitation by ensuring broader geographical representation, thereby strengthening the reliability of the findings.

Another limitation was the absence of including the residence of companion animal owners, as animals living in rural areas are more exposed to fleas, which may influence the awareness of the owners regarding these ectoparasites and their management.

## Conclusions

This study provides valuable insight into the KP of companion animal owners in Mainland Portugal regarding flea control and flea-borne pathogens, and their management. Many pet owners lacked specific knowledge about pathogens transmitted by fleas, despite general awareness of their role in infection transmission. While common strategies like cleaning and ectoparasite control were reported, the understanding of comprehensive flea control was inconsistent. Veterinarians emerged as primary sources of information on flea and flea-borne pathogens prevention, reflecting their crucial role in educating owners following a One Health approach.

## Supplementary Information

Additional file 1: Supplementary Fig. 1. Questionnaire regarding knowledge and practices of companion animal owners regarding fleas, flea-borne pathogens, and measures for their treatment and prevention

Additional file 2: Supplementary Fig. 2. Protocol implemented for scoring knowledge regarding fleas, knowledge regarding flea-borne pathogensand practicesof pet owners, according to the answers provided in the questionnaire

Additional file 3: Supplementary Fig. 3. Distribution of the number of participants included in the study by NUTS3 region in Mainland Portugal

Additional file 4: Supplementary Fig. 4. Distribution of individual **a** knowledge regarding flea scores, **b** knowledge regarding flea-borne pathogen scores, **c** practices scores.

Additional file 5: Supplementary Table 1. Distribution of participants and knowledge and practices scores by category, for sociodemographic variables

## Data Availability

The datasets generated and analysed during the current study are not publicly available due to confidentiality commitments with the participants, as stated in the consent declaration, but are available from the corresponding author on reasonable request.
