# Supplementary material for: Knowledge and practices of dog and cat owners in Mainland Portugal regarding fleas, flea-borne pathogens, and their management
Source: Parasit Vectors. 2025 Jul 4;18:254. doi: 10.1186/s13071-025-06876-y (PMC12228207; doi:10.1186/s13071-025-06876-y)
Supplement: Supplementary file 1 — Additional file 1: Supplementary Fig. 1. Questionnaire regarding knowledge and practices of companion animal owners regarding fleas, flea-borne pathogens, and measures for their treatment and prevention [file 13071_2025_6876_MOESM1_ESM.pdf]

QUESTIONNAIRE ON FLEAS AND FLEA-BORNE DISEASES

1. Number of dogs and/or cats living with you: __________ dog(s) __________ cat(s)

2. Lifestyle: *(choose 1 option in each species, if applicable)*

DOG *(if applicable)*
☐ Living exclusively indoors
☐ Living indoors and having access / going outdoors (street, yard...)
☐ Living exclusively outdoors

CAT *(if applicable)*
☐ Living exclusively indoors
☐ Living indoors and having access / going outdoors (street, yard...)
☐ Living exclusively outdoors

3. Has (have) your pet(s) ever had fleas? *(choose 1 option)*

☐ Yes ☐ No ☐ I don't know / I don't remember

If you answered YES, please continue to question 3(a). If you answered NO or I DON'T KNOW / DON'T REMEMBER, please jump to question 4.

3(a). How did you know your pet had fleas? *(choose 1 or more options)*

☐ Through the animal's behaviour (e.g. scratching more than usual)
☐ By observing suspicious signs of allergic reaction (e.g. hair loss)
☐ By the observation of fleas on the animal
☐ By observing fleas in the environment (e.g. carpets, car, beds)
☐ Flea bites on members of your household
☐ The veterinary doctor said
☐ Other. Which one? __________________
☐ I don't know / I don't remember

4. Do you deworm your pet(s) against fleas? *(choose 1 option)*

☐ Yes ☐ No ☐ I don't know / I don't remember

If you answered NO please continue to question 4(a). If you answered YES, please jump to question 5. If you answered I DON'T KNOW / DON'T REMEMBER, please jump to question 10.

4(a). If NO, do you have any reason(s) for not deworming your pet(s) against fleas? *(choose 1 or more options)*

☐ Too expensive
☐ The deworming scheme against fleas seems complicated
☐ The veterinary doctor did not recommend
☐ My pet has no fleas
☐ I do not consider it effective
☐ I do not consider it safe
☐ I consider the risk of contact with fleas and associated diseases to be low
☐ Other. Which one? __________________
☐ I don't know / I don't remember

Please jump to question 10

5. Why do you deworm your pet(s) against fleas? *(choose 1 or more options)*

☐ To prevent getting fleas
☐ As a treatment, when I see fleas
☐ Because the veterinary doctor recommends
☐ Because I heard about it on TV / radio / social media
☐ Other. Which one? __________________
☐ I don't know / I don't remember

6. How often do you deworm your pet(s) against fleas? *(choose 1 or more options)*

☐ Once a month ☐ 3 in 3 months ☐ 4 in 4 months ☐ 6 in 6 months ☐ 8 in 8 months ☐ Once a year
☐ Other. Which one? __________________ ☐ I don't know / I don't remember

7. What type of flea dewormer do you use on your pet(s)? *choose 1 or more options)*

☐ Pipettes (spot-on) ☐ Collar ☐ Pill ☐ Shampoo ☐ Spray

☐ Other. Which one? __________________ ☐ I don't know / I don't remember

8. If you have more than one animal at home, and one of them has fleas, do you deworm all the animals? *(choose 1 option. If not applicable, do not answer)* ☐ Yes ☐ No ☐ I don't know / I don't remember

9. If you have both cats and dogs, do you deworm both? *(choose 1 option. If not applicable, do not answer)*

☐ Yes, with the same frequency ☐ Yes, with different frequencies ☐ No ☐ I don't know / I don't remember

If you answered NO please continue to question 9(a). If you answered YES or I DON'T KNOW / DON'T REMEMBER, please jump to question 10.

9(a). If you answered NO, what is the reason for not deworming both? *(choose 1 or more options)*

☐ Only the dog(s) have fleas ☐ Only the cat(s) have fleas ☐ The cat(s) do not go outside

☐ Other. Which one? __________________ ☐ I don't know / I don't remember

10. Indicate which of the follow aspects are present or characterise adult fleas. *(choose 1 or more options)*

☐ Rounded body ☐ Flattened body ☐ Brown colour ☐ White colour ☐ They fly ☐ They jump

☐ They eat blood ☐ They eat dead skin ☐ They eat meat (muscle)

☐ They measure more than 1cm ☐ They measure less than 1cm ☐ I don't know / I don't remember

11. In your opinion, what is the most common way for animals to get fleas? *(choose 1 or more options)*

☐ Through the environment, at home (carpets, beds, floors) ☐ Through the environment, outside the house (street, garden, park) ☐ Through another animal ☐ Through people

☐ Other. Which one? __________________ ☐ I don't know / I don't remember

12. In your opinion, when are fleas active? *(choose 1 or more options)*

☐ All year round ☐ Only in winter ☐ Only in spring ☐ Only in summer

☐ Only in autumn ☐ From spring to autumn

☐ Other. Which one? __________________ ☐ I don't know / I don't remember

13. In your opinion, can the fleas that parasitize the dog/cat also parasitize you? *(choose 1 option)*

☐ Yes ☐ No ☐ I don't know / I don't remember

14. Did you know that fleas can transmit diseases? *(choose 1 option)*

☐ Yes ☐ No ☐ I don't know / I don't remember

If you answered NO or I DON'T KNOW / DON'T REMEMBER, please jump to question 19. If you answered YES, please continue to question 15.

15. In your opinion, how does the transmission of an infectious agent from the flea to the animal occur?
(choose 1 or more options)

☐ Through the flea's bite ☐ Through the flea's faeces ☐ Through ingestion of the flea

☐ Other. Which one? __________________ ☐ I don't know / I don't remember

16. In your opinion, how can a human be infected by flea-borne agents? *(choose 1 or more options)*

☐ By the animal's scratch ☐ By the animal's bite ☐ By contact with the animal ☐ Through the flea's bite

☐ Through the flea's faeces ☐ Through ingestion of the flea

☐ Other. Which one? __________________ ☐ I don't know / I don't remember

17. Indicate diseases that you think are transmitted by fleas. *(please list up to 3 examples)*

____

18. What is/are your usual source(s) regarding fleas and flea-borne diseases? *(choose 1 or more options)*

☐ Veterinary doctor ☐ Physician ☐ Television and radio ☐ Magazines and newspapers ☐ Internet / social media

☐ Friends and family ☐ Pharmacy ☐ Education/Profession

☐ Other. Which one? _________________ ☐ I don't know / I don't remember

19. If your pet has fleas, do or would you do any of the following practices to control the infestation? *(choose 1 or more options)*

☐ I clean and vacuum the house / animal's resting places

☐ I apply insecticidal products in the animal's home / resting places

☐ I use safety nets on windows and doors

☐ I clean and vacuum the car

☐ Brush the animal with a comb indicated for the removal of fleas

☐ I keep the animals indoors from sunset until sunrise

☐ I ask the veterinarian for advice on flea prevention and control

☐ Other. Which one? ____

☐ I don't know / I don't remember

20. What is your age? _______ years **21. What is your sex?** ☐ F ☐ M ☐ I prefer not to say

22. What is your municipality of residence? ____

23. What are your academic qualifications? *(choose 1 option)*

☐ Primary Education (1st to 4th grade)
☐ Elementary Education (5th to 9th grade)
☐ High School (10th to 12th grade)
☐ Higher education (bachelor, master, doctorate)
☐ None of the above
☐ I don't know / I don't remember

This questionnaire ends here.

We thank you for your participation.
