# Supplementary material for: Knowledge and practices of dog and cat owners in Mainland Portugal regarding fleas, flea-borne pathogens, and their management
Source: Parasit Vectors. 2025 Jul 4;18:254. doi: 10.1186/s13071-025-06876-y (PMC12228207; doi:10.1186/s13071-025-06876-y)
Supplement: Supplementary file 2 — Additional file 2: Supplementary Fig. 2. Protocol implemented for scoring knowledge regarding fleas, knowledge regarding flea-borne pathogensand practicesof pet owners, according to the answers provided in the questionnaire [file 13071_2025_6876_MOESM2_ESM.docx]

**Supplementary figure 2**

Protocol implemented for scoring knowledge regarding fleas (Kf), knowledge regarding flea-borne diseases (Kd) and practices (Pra) of pet owners, according to the answers provided in the questionnaire.

1. **Knowledge regarding fleas (Kf) score**

Total Kf score for each pet owner obtained by adding individual question scores

Higher scores representing higher level of knowledge

(Minimum 0 – Maximum 4)

| Question description | Answer(s) selected | Score |
| --- | --- | --- |
| **Indicate which of the follow aspects are present or characterise adult fleas** | All the correct answers (and not others): "Flattened body", "Brown colour", "They jump", "They eat blood", "They measure less than 1cm" | 1 |
|  | All the correct answers and other responses | 0.75 |
|  | Some correct answers and none of the other responses | 0.5 |
|  | Some correct answers and other responses | 0.25 |
|  | Only other responses OR No answer | 0 |
| **In your opinion, what is the most common way for animals to get fleas?** | All the correct answers: "Through the environment, at home", "Through the environment, outside the house" | 1 |
|  | All the correct answers and "Through another animal" | 0.8 |
|  | All the correct answers and other responses OR Some correct answers and none of the other responses | 0.6 |
|  | Some correct answers and "Through another animal" | 0.4 |
|  | Some correct answers and other responses OR Only "Through another animal" | 0.2 |
|  | Only other responses OR No answer | 0 |
| **In your opinion, when are fleas active?** | “All year round” | 1 |
|  | “From Spring to Autumn” | 0.5 |
|  | Other responses OR No answer | 0 |
| **In your opinion, can the fleas that parasitize the dog/cat also parasitize you?** | “Yes” | 1 |
|  | “No” OR “I don't know / I don't remember” | 0 |

1. **Knowledge regarding flea-borne diseases (Kd) score**

Total Kd score for each pet owner obtained by adding individual question scores

Higher scores representing higher level of knowledge

(Minimum 0 – Maximum 4)

| Question description | Answer(s) selected | Score |
| --- | --- | --- |
| **Did you already know that fleas can transmit diseases?** | “Yes” | 1 |
|  | “No” OR “I don't know / I don't remember” | 0 |
| **In your opinion, how does the transmission of an infectious agent from the flea to the animal occur?** | All the correct answers: “Through the flea’s bite”, “Through the flea’s faeces”, “Through ingestion of the flea” | 1 |
|  | 2/3 correct answers | 0.75 |
|  | 1/3 correct answers | 0.25 |
|  | Other responses OR No answer | 0 |
| **In your opinion, how can a human be infected by flea-borne agents?** | All the correct answers: "Through the flea’s bite", "Through the flea’s faeces", "Through ingestion of the flea" | 1 |
|  | All the correct answers and "By the animal's scratch” and/or “By the animal's bite” | 0.8 |
|  | All the correct answers and other responses OR Some correct answers and none of the other responses | 0.6 |
|  | Some correct answers and "By the animal’s scratch" and/or "By the animal’s bite" | 0.4 |
|  | Some correct answers and other responses OR Only "By the animal’s scratch" and/or "By the animal’s bite" | 0.2 |
|  | Other responses OR No answer | 0 |
| **Indicate diseases that you think are transmitted by fleas.** | All the diseases mentioned are transmitted by fleas | 1 |
|  | Most of the diseases mentioned are transmitted by fleas | 0.75 |
|  | Half of the diseases mentioned are transmitted by fleas | 0.5 |
|  | A minority of the diseases mentioned are transmitted by fleas | 0.25 |
|  | Does not answer or none of the diseases mentioned are transmitted by fleas | 0 |

1. **Practices (Pra) score**

Total Pra score for each pet owner obtained by adding individual question scores

Higher scores representing higher level of protective practices

(Minimum 0 – Maximum 7)

| Question description | Answer(s) selected | Score |
| --- | --- | --- |
| **Do you deworm your pet(s) against fleas?** | “Yes” | 1 |
|  | “No” OR “I don't know / I don't remember” | 0 |
| **If you have more than one animal at home, and one of them has fleas, do you deworm all the animals?** | “Yes” | 1 |
|  | “No” OR “I don't know / I don't remember” | 0 |
| **If you have both cats and dogs, do you deworm both?** | “Yes, with the same frequency” | 1 |
|  | “Yes, with different frequencies” | 0.5 |
|  | “No” OR “I don't know / I don't remember” | 0 |
| **If your pet has fleas, do or would you do any of the following practices to control the infestation?** | All the following answers: “I clean and vacuum the house / animal's resting places”, “I apply insecticidal products in the animal's home / resting places”, “I clean and vacuum the car”, “Brush the animal with a comb indicated for the removal of fleas” | 4 |
|  | Three of the above | 3 |
|  | Two of the above | 2 |
|  | One of the above | 1 |
|  | None of the above OR No answer | 0 |
