# Supplementary figures and images for: Knowledge and practices of dog and cat owners in Mainland Portugal regarding fleas, flea-borne pathogens, and their management
Source: Parasit Vectors. 2025 Jul 4;18:254. doi: 10.1186/s13071-025-06876-y (PMC12228207; doi:10.1186/s13071-025-06876-y)

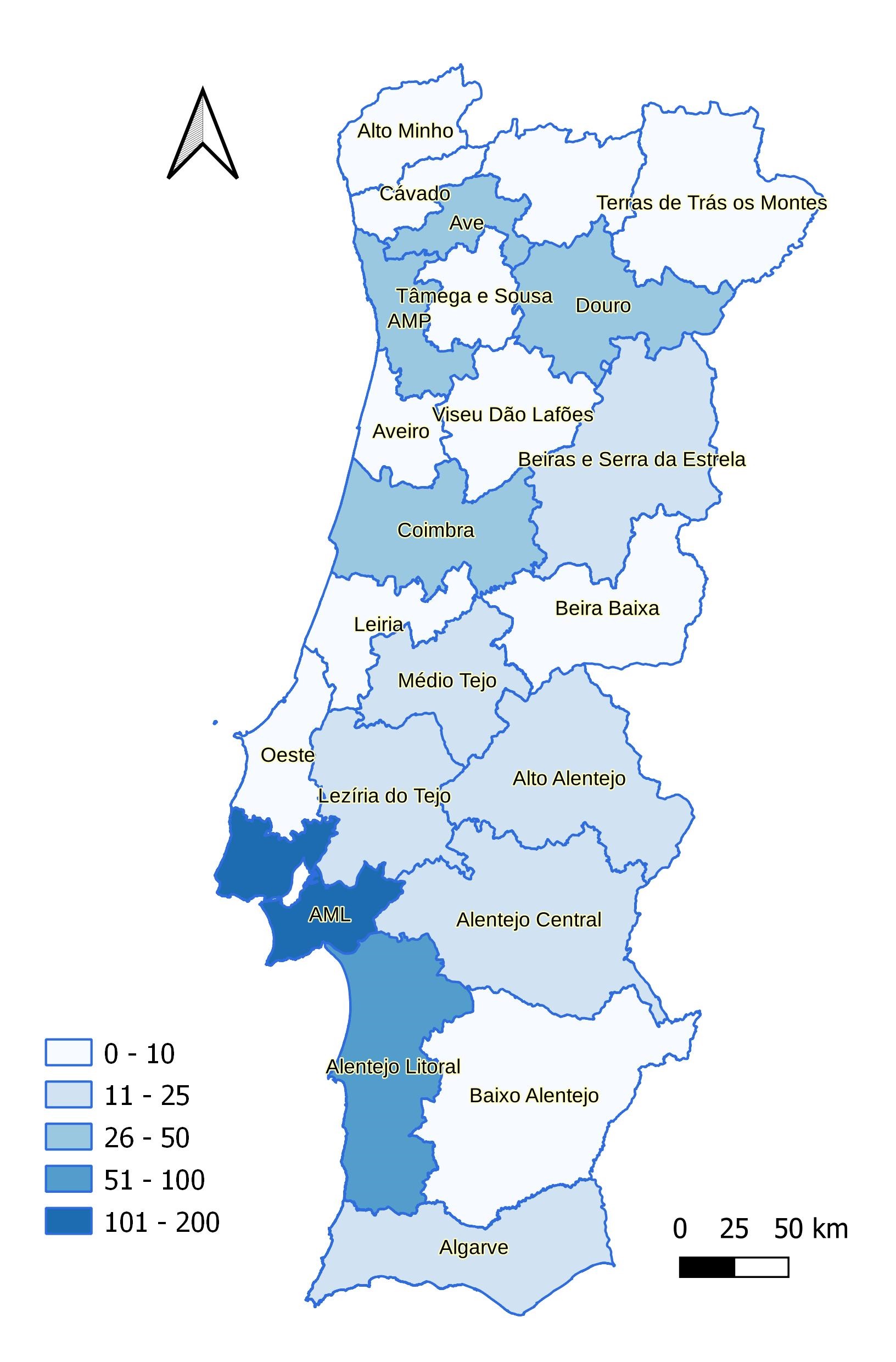

Supplement: Supplementary file 3 — Additional file 3: Supplementary Fig. 3. Distribution of the number of participants included in the study by NUTS3 region in Mainland Portugal [file 13071_2025_6876_MOESM3_ESM.jpeg]

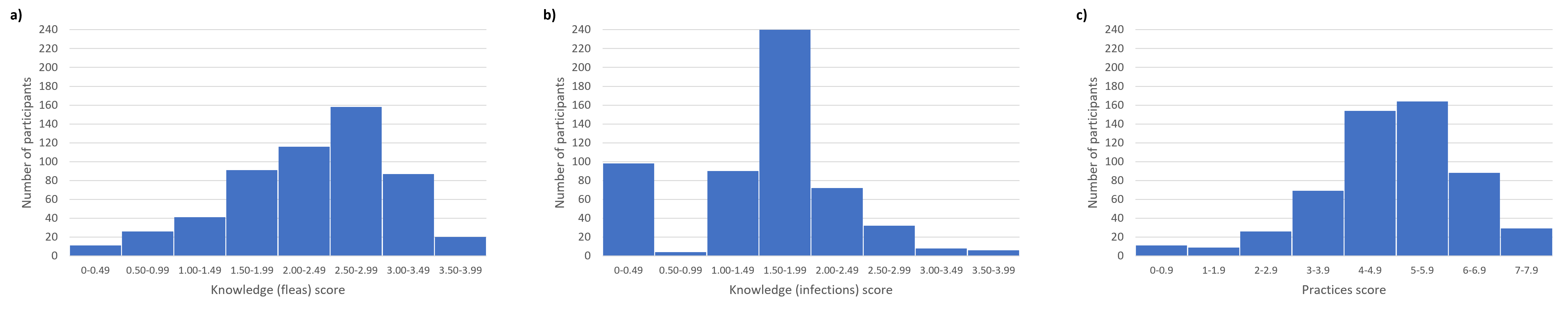

Supplement: Supplementary file 4 — Additional file 4: Supplementary Fig. 4. Distribution of individual a knowledge regarding flea scores, b knowledge regarding flea-borne pathogen scores, c practices scores. [file 13071_2025_6876_MOESM4_ESM.png]
