# Supplementary material for: Knowledge and practices of dog and cat owners in Mainland Portugal regarding fleas, flea-borne pathogens, and their management
Source: Parasit Vectors. 2025 Jul 4;18:254. doi: 10.1186/s13071-025-06876-y (PMC12228207; doi:10.1186/s13071-025-06876-y)
Supplement: Supplementary file 5 — Additional file 5: Supplementary Table 1. Distribution of participants and knowledge and practices scores by category, for sociodemographic variables [file 13071_2025_6876_MOESM5_ESM.docx]

**Supplementary Table 1.** Distribution of participants and knowledge and practices scores by category, for sociodemographic variables.
